# Are smokers scared by COVID-19 risk? How fear and comparative optimism influence smokers’ intentions to take measures to quit smoking

**DOI:** 10.1371/journal.pone.0260478

**Published:** 2021-12-07

**Authors:** Hue Trong Duong, Zachary B. Massey, Victoria Churchill, Lucy Popova

**Affiliations:** 1 Department of Communication, Georgia State University, Atlanta, GA, United States of America; 2 School of Journalism, University of Missouri, Columbia, MO, United States of America; 3 School of Public Health, Georgia State University, Atlanta, GA, United States of America; University of Haifa, ISRAEL

## Abstract

Research suggests that smoking may compound the risk of serious health problems to smokers who contract COVID-19. This study examines whether and how exposure to news stories reporting the severe COVID-19 risk to smokers may influence smokers’ emotional responses (fear, anxiety, and sadness) and intentions to take measures to quit smoking. Current smokers in the US participated in an online experiment (*N* = 495) and were randomized to read smoking risk news stories or news stories reporting the combined risk of smoking and COVID-19. We found that combined risk news stories lead to participants feeling more fearful and sadder than when they viewed smoking risk news stories (*M* = 5.74; *SD* = 2.57 *vs*. *M* = 5.20; *SD* = 2.74; *p* < .05). Fear fully mediated the effect of news exposure on intentions to take measures to quit smoking (*ß* = .09; *SE* = 05; 95% CI [.010, .200]). Moreover, moderated-mediation analyses revealed that the mediating effect of fear was conditioned on the levels of comparative optimism, such that the association between fear and intentions to take measures to quit smoking was only significant among smokers whose comparative optimism was at the mean score (*ß* = .16; *SE* = 05; 95% CI [.071, .250]), and for those whose comparative optimism was high (*ß* = .27; *SE* = .06; 95% CI [.146, .395]). These results suggest that fear of the pandemic and optimism might play important roles in predicting and explaining the association between exposure to news stories and intentions to take measures to quit smoking. Messages about heightened risk of COVID-19 complications for smokers that increase fear might be an effective strategy to motivate smokers to quit. Such messages should be used to turn the adversity of COVID-19 pandemic into an intervention opportunity to reduce tobacco-related disease.

## Introduction

Tobacco smoking results in inflammatory processes in the lungs, causing many respiratory diseases [1, 2]. Smoking also damages the immune system, making smokers more susceptible to infectious diseases [3, 4]. The Coronavirus disease 2019 (COVID-19) is a viral disease characterized by severe acute respiratory syndrome, which has caused a worldwide pandemic. Smoking can compound the risk of serious health problems to smokers who contract COVID-19. Research has found that smokers are at a higher risk of experiencing severe illness from COVID-19 than non-smokers [5–7].

Communicating about risks of smoking has been effective at motivating smokers to quit, and messages about novel risks might be particularly effective for some smokers [8–10]. Researchers proposed that the COVID-19 pandemic may provide an impetus for smokers to quit because smokers can be vulnerable to severe COVID-19 illness [11–13]. Some recent studies found higher smoking cessation rates and greater intentions to quit during the pandemic than before [14, 15]. These behavioral intentions changes might be caused by smokers’ negative emotional responses to the pandemic threat [15]. This suggests that emotional responses to possible severe COVID-19 complications might be a source of motivation for smokers leading to greater intentions to quit.

Although researchers have recommended that anti-smoking interventions consider communicating the ramifications of COVID-19 to smokers [13, 16], little research has examined whether and how exposure to information about the severe COVID-19 risk to smokers might motivate behavioral change, above and beyond exposure to general smoking risk information (for an exception, see [11]). The current study addresses this gap by investigating the relative effect of combined COVID-19 and smoking risk information compared to smoking risk information only on smokers’ fear, anxiety, and sadness, to investigate how those emotions are associated with intentions to quit smoking. We examined these emotions as mediators because past research indicates that negative emotions serve as the mechanism through which anti-smoking messages affect quitting intentions [17–19]. In addition, we examined the role of comparative optimism—a belief that one’s own risk is lower than the risk of comparable others [20]. Comparative optimism can be particularly prominent during the COVID-19 pandemic because of people’s need to mentally and physically cope with the pandemic [21]. Together, the purpose of this study is to examine whether exposure to severe COVID-19 risk information could elicit emotional responses that affect intentions to take measures to quit smoking among smokers with different levels of comparative optimism.

### Emotional responses to COVID-19

An infectious pandemic presents an acute threat [22]. Research shows that exposure to information about such an acute threat commonly arouses fear and anxiety [23, 24]. Moreover, observation of numerous others’ suffering and deaths during an infectious outbreak tends to evoke sadness [25]. These emotions can be particularly relevant to smokers because smoking-related COVID-19 disease information possibly conjures up the prospect of severe complications. Research has found that exposure to smoking disease information may arouse similar emotions for smokers [26]. However, smoking disease likely represents a chronic threat to smokers due to their prolonged exposure to anti-smoking information, and thus, the threat might be perceived as normal and distant in time [9, 27]. Smoking also results in noncommunicable disease that smokers might perceive as controllable. Therefore, smokers’ levels of emotional reactions to the risk of an infectious outbreak might be higher than that of smoking disease.

During a disease outbreak, the media tends to use emotional language [28] and portray the deaths of those who suffer from the disease [29]. News about this COVID-19 pandemic has similarly used emotional language when describing death counts and showing images of dying patients [30]. The media have also reported risk factors for COVID-19 complications, including greater risk for severe disease progression among smokers [31]. In addition, numerous news stories have reported on tobacco users who died from COVID-19. Exposure to COVID-19 news stories presenting the severe risk of COVID-19 complications might evoke negative emotions for smokers because the news is particularly relevant to them. In this study, we referred to news that contained only smoking risk as smoking risk stories and news reporting COVID-19 severe risk to smokers as combined risk news stories. We hypothesize:

*H1*: Compared to the smoking risk news stories, the combined risk news stories will evoke significantly higher fear (*H1a*), anxiety (*H1b*), and sadness (*H1c*) in smokers.

### Emotions as mediators of intentions to quit smoking

Emotions are defined as evaluative and valenced mental states that served to help humans adapt to their surrounding environments [32]. The Appraisal Tendency Framework (ATF, [33]) posits that each emotion is defined by a core appraisal theme that provides information about specific harms or benefits, which subsequently influence a specific course of action [34]. Six cognitive dimensions that underpin the appraisal patterns of emotions have been identified: certainty, pleasantness, attentional activity, control, anticipated effort, and responsibility [35]. For example, fear is associated with high uncertainty and low personal control over a threat and, therefore, likely triggers actions to perform behaviors to avert the threat [32]. Anxiety is associated with uncertainty related to existential threats and accompanies the action tendency to reduce uncertainty [33]. Sadness is characterized by appraisals of experiencing irrevocable loss [33] and thus, accompanies the action tendencies to change circumstances, such as seeking rewards [35]. Thus, the ATF posits that different emotions may lead to different appraisal and behavioral tendencies [36].

Research has shown that emotions tend to have an impact on smokers’ behaviors. For example, studies found that negative emotions (e.g., fear, sadness, guilt) evoked by anti-smoking messages motivated smokers to quit smoking [17, 18, 37]. A recent study using an online experiment with smokers found that messages about the harms of smoking and COVID-19 increased smokers’ negative emotional reactions [11]. These studies, however, utilized a valence-based approach as they theorized that emotional valence dimensions (i.e., positiveness or negativity) influence behavioral intentions. The ATF, however, argues that emotions have different associations with cognitive appraisals and themes, which determine individuals’ judgments and choices [36]. Using the ATF, Yang et al. [26] found that messages communicating the risk of smoking cigarettes aroused higher fear, guilt, disgust, hope, and anger. The first four emotions were associated with higher intentions to quit smoking, seek quit help, use nicotine replacement therapy (NRT), switch to e-cigarettes, and use e-cigarettes exclusively. Anger, however, was related to weaker intentions to quit smoking. Thus, although fear and anger share a negative valence, they may activate different action tendencies toward smoking cessation intentions. However, research using the ATF to examine the effect of specific emotions on smokers’ behavioral intentions in the context of COVID-19 is lacking.

While the literature has documented the effects of fear induced from exposure to warning labels and anti-smoking messages on smoking cessation [37–40], research has not investigated how fear, anxiety, and sadness might concomitantly influence smokers’ behavioral change during the COVID-19 pandemic. In this study, we focused on smokers’ intentions to quit smoking (hereafter referred to as behavioral intentions). These intentions include specific behaviors, such as reducing cigarette consumption, seeking quit help, and using NRT. These behaviors represent specific and easily accessible pathways to successfully quit smoking [41]. Consistent with the ATF framework and previous research findings, we hypothesize:

*H2*: Fear (*H3a*), anxiety (*H3b*), and sadness (*H3b*) will mediate the association between exposure to the combined risk news stories and behavioral intentions.

### The moderating role of comparative optimism

People tend to believe that they are less likely than their peers to experience negative events—a phenomenon referred to as comparative optimism [20, 42, 43]. Comparative optimism is common in health-related contexts and is motivated by self-enhancement (individuals’ focus on what they want to happen rather than on what might happen), self-presentation (individuals’ need to establish and maintain a positive personal image), or personal control (individuals’ tendency to believe that they are better than others to control an outcome) [20]. Comparative optimism is ubiquitous across different ages, cultural groups, and risk contexts [44–46]. Related to COVID-19, research has found that individuals believe they are less likely to be infected than other individuals of the same age and gender [47, 48].

There are two lines of theoretical propositions about the effect of comparative optimism on health behavior. The first is that comparative optimism negatively affects protective behavior because individuals’ underestimation of a health risk may impede their adoption of healthy behavior [49, 50]. For instance, smokers who are optimistic about having low chances of developing lung cancer from smoking are less likely to plan to quit smoking than smokers who are not [51]. Additionally, research found that the association between smokers’ perceived cancer risk and their intentions to seek cancer prevention information is weakened among smokers with higher comparative optimism [52]. During an infectious outbreak, researchers found that the effect of talking about the H1N1 pandemic on preventive behaviors is only significant for individuals with low or moderate, rather than high, comparative optimism [53].

In contrast, the second line of theorization posits a positive association between comparative optimism and health behavior because individuals taking preventive measures may form a perception of self-invulnerability. This theoretical perspective suggests that comparative optimism is a relatively accurate perception, reflecting individuals’ preventive behavior [50]. Thus, researchers argue that comparative optimism can be informed by dispositional optimism, which is a general predisposition toward an optimistic outlook to external events [54]. Dispositional optimists generally assess their risk as lower than others [55]. This form of optimism is particularly enacted when people need to cope with an extreme traumatic event [56]. Thus, comparative and dispositional optimism can be positively associated despite being two distinct phenomena [57]. In support of this theoretical perspective, a meta-analysis revealed that in extreme traumas, individuals likely adjust their optimistic expectancies to engage in coping strategies and managing emotions to move on [56]. The beginning stages of the COVID-19 pandemic provide an extreme health risk circumstance, and following this line of research, dispositional optimism may inform comparative optimism to activate individuals’ coping mechanisms. Researchers examining optimism during the deadliest stages of the COVID-19 outbreak in Italy (March-April 2020) found that individuals with higher dispositional optimism also possessed higher comparative optimism related to COVID-19 [21]. The researchers explained that dispositional optimism might prevail and dictate comparative optimism under such an extreme threatening and stressful condition. So far, this research finding has been the most relevant to the current study because it shares the same health risk topic and similar context (i.e., the COVID-19 outbreak in its most fearful stages with intensive media coverage on deaths and hospitalization [23]). Therefore, we conjecture that comparative optimism might function similarly to dispositional optimism, where comparative optimism would be associated with positive outcomes (behavioral intentions to quit smoking) for smokers experiencing negative emotions. Thus, we hypothesize:

H3: Comparative optimism will moderate the mediation effects of fear (*H3a*), anxiety (*H3b*), and sadness (*H3c*) on behavioral intentions, such that the mediation effects will be enhanced among smokers with higher comparative optimism and attenuated among smokers with lower comparative optimism.

## Methods

### Participants and procedures

This study was part of a larger online experiment examining news exposure and smokers’ reactions, which had three treatment conditions (COVID-19 risk news stories, smoking risk news stories, combined COVID-19 and smoking risk news stories) and a control condition (exposure to neutral news stories such weather forecast). For this study, we analyzed data from participants in the smoking risk condition and the combined risk condition only (*N* = 495). Current smokers were recruited by the market research company Toluna (www.toluna-group.com) and participated in this study in August 2020. Toluna used multiple online recruitment channels (e.g., web banners, website referrals, affiliate marketing, pay-per-click) to recruit eligible participants. Eligibility criteria were being a current smoker (smoked 100 cigarettes in their lifetime and now smoked every day or somedays), 18 years old or above, and residing in the US. Participants completed an electronic informed consent. All protocols were approved by a University’s Institutional Review Board. A flowchart of participants is included in Appendix A in S1 Appendix. Data are available at https://scholarworks.gsu.edu/sph_datasets/2/.

Participants provided information on their demographics, psychological distress, self-rated health status, heaviness of smoking, and pre-existing intentions to quit smoking via the online questionnaire. Participants were then randomized to one of the four experimental conditions in a 1:1:1:1 ratio using a least-fill randomiser function. Immediately after viewing the news story, participants completed measures of emotions, behavioral intentions, and comparative optimism, followed by prior knowledge of COVID-19 risk to smokers. Finally, participants viewed a debriefing page that explained that the messages were for research purposes only and have not been approved by any state or federal government agency. The page also provided information on the more severe COVID-19 for smokers, which was adapted from the Stanford Tobacco Prevention Toolking [58], and a quit line number and a link to a smoking cessation website [59].

### Stimulus materials

We created five news stories for each experimental condition to address the potential case-category confounding problems in experimental research with stimulus materials [60]. Participants in the same treatment conditions were randomly assigned to view one news story from a set of five news stories. All news stories were created by modifying real news stories collected from several online news websites, such as *ABC*, *New York Times*, *Fox News*, and *Los Angeles Times*. In the smoking risk condition, the news topic was the risk of lung disease associated with smoking. In the combined risk condition, the news topic concerned the severe outcomes of COVID-19 disease to smokers. The news stories concluded with advice to quit smoking to prevent disease.

All news stories were approximately equal in length (ranging between 220–250 words). To enhance ecological validity, the news stories contained photos similar to the photos in the original news stories. As the COVID-19 pandemic has been linked to heated debates centering on American politics and media credibility [61], the news stories made no reference to any sources nor journalists to control for confounding effects (see Appendix B in S1 Appendix for a set of news stimulus samples).

### Measures

Fear, anxiety, and sadness were measured by asking participants to report how much they felt afraid, worried, and sad when they viewed the news stories (1 = *not at all*, 9 = *extremely*, fear: *M* = 5.46; *SD* = 2.67; anxiety: *M* = 5.86; *SD* = 2.48; sadness: *M* = 6.35; *SD* = 2.55) [26]. Behavioral intentions were assessed with three items asking participants to report how likely in the next 6 months they would 1) reduce the number of cigarettes they smoke in a day; 2) seek counselling support to help them quit smoking; and 3) use nicotine gum, nicotine patch, or other forms of NRT (1 = *not at all likely*, 9 = *extremely likely*, [62]) Responses to these items were averaged into a composite score (*α* = .75; *M* = 5.24; *SD* = 1.16). Comparative optimism was assessed by subtracting participants’ scores estimating their chances of catching COVID-19 (*M* = 49.63; *SD* = 28.02) from the chances that they estimated for another smoker of the same sex and health status (*M* = 53.83; *SD* = 26.35). Participants dragged a slide bar to estimate the risk for themselves and for the other smoker (ranging between *0% chance*, *100% chance*). Higher scores represented higher comparative optimism (higher risk for others compared to one own’s risk; *M* = 4.19; *SD* = 23.38).

Potential covariates included sex, age, race, education, income, prior knowledge of increased COVID-19 risk to smokers, psychological distress, self-rated health status, heaviness of smoking, and pre-existing intentions to quit smoking (i.e., participants’ intentions to quit smoking prior to their exposure to the news stimulus, see Table 1). Specifically, prior knowledge of COVID-19 risk to smokers was measured by asking participants to report how much they had heard about the risk of the coronavirus for smokers prior to the study (1-*nothing at all*, 4-*a lot*; *M* = 2.48; *SD* = .99). Psychological distress [63] was measured by asking participants to report how often they felt very sad, nervous, restless, hopeless, worthless, and that everything was an effort (1-*all the time*, 5-*none of the time*, *α* = .91; *M* = 3.26; *SD* = 1.10). Self-rated health status [64] was assessed by asking participants to describe their health in general (1-*excellent*, 5-*poor; M* = 2.59; *SD* = 1.10). Heaviness of smoking [65, 66] was assessed through participants’ report how soon after they wake up in the morning that they usually smoked their first cigarette (0-*after 60 minutes*, 3-*within 5 minutes*) and how many cigarettes per day they smoke on average (0- ten or less, 3–31 or more, *r* = .38, *p* < .01).

**Table 1:** Participants’ demographics.

	Overall (*N* = 495), %	Smoking risk condition (*n* = 252), %	Combined risk condition (*n* = 243), %
Sex			
Male	53	50	56
Female	47	50	44
Age			
18–29	30.3	31	29.6
30–44	32.3	33.7	30.9
45–59	22	20.2	23.9
60+	15.4	15.1	15.6
Race			
White	70.3	67	73.7
Black	15.6	18.7	12.3
Asian	3.8	4	3.7
American Indian	2.8	3.6	2
More than one race	3.8	3.2	4.5
Other	3.6	1.7	.5
Education			
Less than high school	12.5	12.3	12.8
High school	25.5	27	23.9
Some college	22.8	25.8	19.8
Bachelor’s degree or higher	39.2	24.9	43.6
Annual Income			
< $25,000	26	25	27
$25,000 –$59,000	28	31	25
$60,000+	46	44	48
Heaviness of smoking^a^			
Low	30	33	26
Medium	64	60	67
High	6	7	6
Prior knowledge of COVID-19 Heard about the risks of the coronavirus for smokers			
Nothing at all	19	23	14
A little bit	33	36	30
A moderate amount	30	26	35
A lot	18	16	21
Had been infected with COVID-19			
Yes	12.9	12.3	13.6
No	82.2	81.7	82.7
Maybe	4.8	6	3.7

Note: There were no significant differences between conditions on participant characteristics.

^a^ Heaviness of smoking was calculated by summing the numbers for two questions: time to first cigarette (within 5 minutes [3 points], 6–30 minutes [2 points], 31–60 minutes [1 point], and over 60 minutes [0 points]) and number of cigarettes per day (10 or less [0 points], 11–20 [1 point], 21–30 [2 points], and 31 or more [3 points]) and categorized into low (0–1), medium (2–4), and high (5–6) [88].

### Data analysis

Because our manipulation focused on the absence and presence of news content that included risk information, which were considered intrinsic message features, manipulation checks were not necessary [67]. This methodological approach has been adopted in prior tobacco communication research [68–70]. Data were analyzed using SPSS version 27. There were no significant differences between conditions on gender, age, race, education, income, psychological distress, self-rated health status, heaviness of smoking, prior knowledge of COVID-19 risks to smokers, and pre-existing intentions to quit smoking (*ps* > .05). To test *H1*, independent *t*-tests were conducted to compare fear, anxiety, and sadness between participants in the smoking risk condition and those in the combined risk condition. To test *H2* and *H3*, Hayes’ PROCESS macro v.3.4.1 model 4 and model 14 were used [71]. Exposure conditions served as the independent variable (smoking risk condition = 0; combined risk condition = 1), the three negative emotions (fear, sadness, and anxiety) as the mediators, comparative optimism as the moderator, and intentions to reduce smoking as the dependent variable. To decide on the inclusion of covariates in the model, we followed Pocock et al.’s [72] recommendation to exclude baseline covariates that are weakly (less than *r* = .3) correlated with the outcome (behavioral intentions). Among the potential baseline covariates, only pre-existing intentions to quit smoking was correlated with the outcome (*r* = .34) and were therefore included as a covariate in the model. The indirect effects were considered significant if the 95% bias-corrected confidence intervals (CIs) obtained through bootstrapping procedures with 5,000 resamples did not include zero.

## Results

### Exposures to COVID-19 risk news and emotions

Table 1 reports participants’ demographics. Table 2 showed descriptive statistics and correlations among variables. *H1a-c* stated that smokers who viewed the combined risk news stories would feel more fearful, anxious, and sad than smokers viewing the smoking risk news stories. Smokers in the combined risk condition reported higher fear (*M* = 5.74; *SD* = 2.57 vs. *M* = 5.20, *SD* = 2.74, *t*(493) = -2.267, *p* < .05) and higher sadness (*M* = 7.07; *SD* = 2.18 vs. *M* = 5.65, *SD* = 2.68, *t*(479) = -6.473, *p* < .001) than those in the smoking risk condition. However, results showed no difference in anxiety (*M* = 6.06; *SD* = 2.40 vs. *M* = 5.66, *SD* = 2.54; *t*(493) = -1.792, *p* = .07). Thus, *H1a* and *H1b* were confirmed while *H1c* was not.

**Table 2:** Descriptive statistics and correlations among variables.

	1	2	3	4	5	6
1. Fear	-					
2. Anxiety	.68**					
3. Sadness	.54**	.55**				
4. Comparative optimism	-.04	.02	.01			
5. Behavioral intentions	.34**	.32**	.23**	.02		
6. Pre-existing intentions to quit smoking	.13**	.16**	.07	.08	.34**	-
Mean	5.46	5.86	6.35	4.19	5.24	2.65
SD	2.67	2.48	2.55	23.38	2.16	1.28

Note: All variables were measured on 1(not at all) to 9 (extremely) scale with the exception of comparative optimism (0–100) and pre-existing intentions to quit smoking (1–5).

### Indirect effect of exposure on behavioral intentions

**Fig 1:**
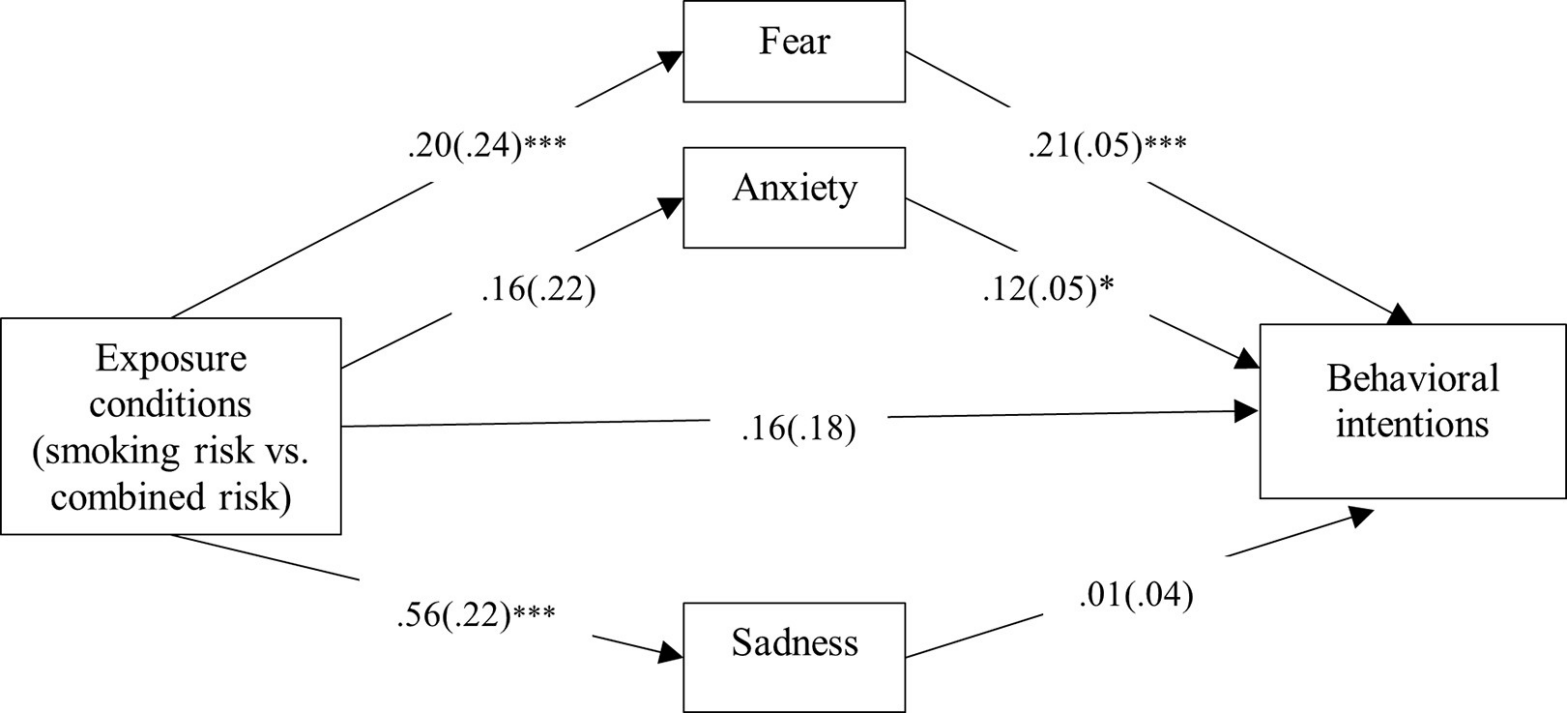
Indirect effects of exposure to news stories on behavioral intentions. *p < .05; ***p < .001.

*H2a-c* hypothesized that fear, anxiety, and sadness would mediate the association between exposure to the news stories and behavioral intentions. Results showed that while fear fully mediated the effect of news exposure on behavioral intentions (*ß* = .09; *SE* = .05; 95% CI [.010, .200]), anxiety (*ß* = .04; *SE* = .03; 95% CI [-.008, .119]) and sadness did not (*ß* = .09; *SE* = .05; 95% CI [-.110, .143]). Thus, results confirmed *H2a*, but not *H2b* and *H2c*. Fig 1 illustrates the mediation model.

### Conditional effect of comparative optimism

**Fig 2:**
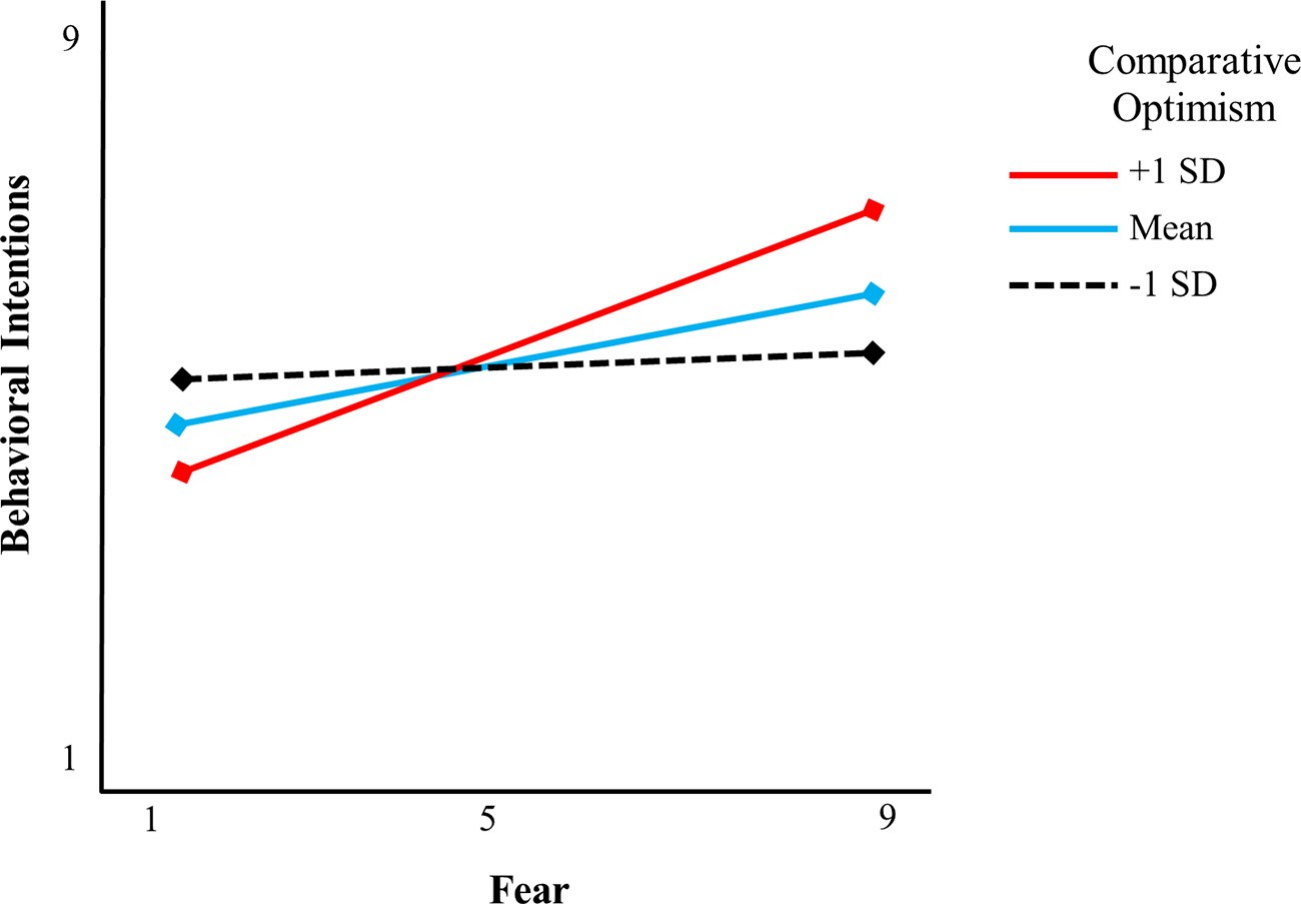
Interactions between fear and comparative optimism for current smokers on behavioral intentions.

*H3a-c* stated that comparative optimism would moderate the mediating effects of fear, anxiety, and sadness on behavioral intentions. Because the test for *H2b* and *H2c* indicated that anxiety and sadness were nonsignificant mediators, *H3b* and *H3c* were not considered further in the moderated-mediation analysis. Results revealed a significant interaction effect between fear and comparative optimism on behavioral intentions (*ß* = .02; *SE* = .01; 95% CI [.001, 009]). Specifically, the mediating effect of fear was nonsignificant for participants whose comparative optimism was low (one standard deviation below the mean score) (*ß* = .05; *SE* = .06; 95% CI [-.086, .186]). However, this mediating effect was significant for participants whose comparative optimism was at the mean score (*ß* = .16; *SE* = .05; 95% CI [.071, .250]), and for those whose comparative optimism was high (one standard deviation above the mean score) (*ß* = .27; *SE* = .06; 95% CI [.146, .395]). Thus, *H3a* was supported (Fig 2).

### Additional analyses

Additional analyses were conducted to further examine the indirect effects of news exposure on the three specific quitting behaviors: 1) intentions to reduce the number of cigarettes in a day; 2) intentions to seek counselling support to help with quitting smoking, and 3) intentions to use nicotine gum, nicotine patch, or other forms of NRT. All indirect pathways as hypothesized in *H2* were nonsignificant for smokers’ intentions to reduce the number of cigarettes they smoke in a day (fear: *ß* = .02; *SE* = .04; 95% CI [-.051, .103]; anxiety: *ß* = .07; *SE* = .05; 95% CI [-.008, .173]; sadness: *ß* = .12; *SE* = .09; 95% CI [-.047, .304]). While fear mediated the effect of news exposure on intentions to seek counselling support (*ß* = .14; *SE* = .07; 95% CI [.016, .299]), anxiety (*ß* = .06; *SE* = .04; 95% CI [-.009, .160]) and sadness did not (*ß* = -.11; *SE* = .09; 95% CI [-.008, .173]). Similar results were found for intentions to use nicotine gum, nicotine patch, or other forms of NRT, with fear was a significant mediator (*ß* = .13; *SE* = .07; 95% CI [.011, .280]), while anxiety (*ß* = .05; *SE* = .04; 95% CI [-.013, .144]) and sadness were not (*ß* = -.01; *SE* = .09; 95% CI [-.181, .177]).

Moderated-mediation results indicated that fear and comparative optimism had a joint effect on intentions to seek counselling support (*ß* = .01; *SE* = .01; 95% CI [.001, .012]) and intentions to use nicotine gum, nicotine patch, or other forms of NRT (*ß* = .01; *SE* = .01; 95% CI [.001, .011]). More specifically, the mediating effect of fear was nonsignificant for participants whose comparative optimism was low (intentions to seek counselling support: *ß* = .07; *SE* = .07; 95% CI [-.027, .229]; intentions to use nicotine gum, nicotine patch, or other forms of NRT: *ß* = .07; *SE* = .06; 95% CI [-.021, .209]), and was significant for participants whose comparative optimism was at the mean score (intentions to seek counselling support: *ß* = .12; *SE* = .07; 95% CI [.013, .280]; intentions to use nicotine gum, nicotine patch, or other forms of NRT: *ß* = .11; *SE* = .06; 95% CI [.009, .254]), and for those whose comparative optimism was high (intentions to seek counselling support: *ß* = .21; *SE* = .10; 95% CI [.025, .423]; intentions to use nicotine gum, nicotine patch, or other forms of NRT: *ß* = .18; *SE* = .09; 95% CI [.016, .390]).

## Discussion

Smokers tend to have more severe symptoms of COVID-19 compared to non-smokers [6], making the COVID-19 pandemic a pressing time for smokers to quit [12]. Messaging around increased risks of COVID-19 for smokers might be particularly motivating at this time. In this study, we found that compared to news stories about smoking risk only, news stories about combined risk of smoking and COVID-19 elicited greater fear and sadness, and fear, in turn, mediated the impact of the combined risk messages on behavioral intentions related to quitting smoking. We also found that this relationship was significant only among smokers who possess greater comparative optimism about their chances of catching COVID-19 compared to other smokers.

Emotions, particularly negative emotions, have been consistently identified as mechanisms through which anti-tobacco messages, such as pictorial warning labels, influence smokers’ health behaviors [17, 18, 73]. However, past research typically looked at negative emotions as a group, which does not allow for the possibility to decompose the unique impact of each particular negative emotion. Guided by the ATF, we examined the separate mediating roles of fear, anxiety, and sadness in the association between exposure to risk news and behavioral intentions. Fear mediated the effect of exposure to combined risk news stories on intentions to take measures to quit smoking. Although the level of sadness was higher for the combined risk news condition, it failed to mediate the association between news exposure and behavioral intentions. We speculate that these findings can be explained by these emotions’ action tendencies. That is, fear is a self-oriented emotion, which likely motivates smokers to find ways to reduce their fear. While smokers can reduce fear through defensive reactions (e.g., denial, downplaying the risk, [74, 75]) protective reactions such as taking measures to quit smoking is perhaps the most sensible option for smokers in COVID-19 outbreak. Meanwhile, despite the significantly higher level of sadness smokers felt from viewing the news stories about COVID-19 compared to viewing the smoking risk news stories, results indicate that sadness does not motivate smokers to take actions to prevent the risk. According to the ATF, sadness is related to a feeling of irrevocable loss, depicting an empathetic response to others’ suffering. Thus, the action tendency associated with sadness is directed at others rather than focusing on the self. As such, sadness can drive pro-social behaviors to support others, such as providing help and monetary donations [25, 76]. Quitting smoking is mostly a health behavior aiming at improving one’s health and thus is self-oriented, which might explain why sadness did not predict intentions to take measures to quit smoking.

While anxiety was positively associated with the intentions outcome, the two conditions did not elicit significantly different levels of anxiety, and anxiety was not a significant mediator. Fear and anxiety are both responses to a threat, but they have been argued to be separate [77], traced to different brain systems [78]. According to the ATF, anxiety is related to existential threat, particularly to the sense of identity. Both conditions questioned the life choices of smokers, presenting existential threat, and resulting in relatively high levels of anxiety. Fear, in contrast, was different and served as a mediator likely because the levels of immediacy and personal control were different: participants in the smoking news condition have heard about harms of smoking before and many smokers believe they can quit before their smoking becomes a problem; this resulted in a lower level of fear. For the combined risk news stories, the information about COVID-19 was novel, the pandemic was immediate, and smokers might have felt that there is not much they could do to protect themselves; this resulted in a higher level of fear.

In the additional analyses where the three specific quitting behavioral intentions were examined separately, the results were substantively identical to those of the combined analysis for the intentions to seek counseling support and to use NRT. However, fear was not a significant mediator for intentions to reduce the number of cigarettes. Although the three intentions varied together (α = .75), among the three, reducing the number of cigarettes is the easiest and the least “costly” intention since it does not require actual quitting or seeking external help. It is possible that smokers were likely to report intentions for reducing the number of cigarettes as a reaction to a simple reminder that smoking is harmful. However, they needed the impetus of fear to translate the message about harms of smoking during COVID-19 into the more effortful cessation activities (seeking counseling and obtaining NRT).

One important contribution of this study rests with clarifying the role of comparative optimism in explaining the association between fear and behavioral intentions. On average, many smokers in our study exhibited comparative optimism, thinking that their chances of catching COVID-19 are lower than other smokers’ chances. We found that comparative optimism interacted with the smokers’ fear, such that the effect of fear on behavioral intentions was greater for smokers who estimated their own risk as lower than others. Our study, therefore, confirms that the effect of fear on smokers’ behavioral intentions is contingent on their cognitive assessments of comparative risk levels between smokers and their peers. As we mentioned above, smokers can engage in defensive or protective responses to threatening messages. Which one they engage in depends on different factors. For example, according to the Extended Parallel Process Model [79], the choice is determined by the level of efficacy—when a person feels that they can do something to avert the threat, they will engage in a protective response (plan to quit smoking) and when a person feels that there is nothing they can do, they will engage in defensive response (downplay the risk or avoid the message). Our study points that comparative optimism might play similar role to that of efficacy. It is likely that comparative optimism and efficacy are related with people who feel they can control the negative events would perceive these events as less likely to occur [80]. This finding also provides more evidence to the speculation that dispositional optimism might reinforce comparative optimism during the COVID-19 outbreak [21]. This moderation finding is consistent with the literature on dispositional optimism. Positive expectations toward self might motivate intentions to take preventive measures as a coping mechanism to manage fear [56].

Our study is one of the first to examine the combination of smoking and COVID-19 risk messages. One recent study found that tobacco users rated messages combining information on COVID-19 and smoking harms as similarly effective as messages that contained only information on traditional harms of smoking [11]. That study used brief messages in the form of tweets and did not find significant differences in the levels of negative emotions between messages with and without COVID-19 harms. Our study may have shown different results because we used news stories depicting real-life people suffering these diseases, which contained concrete exemplars capable of evoking higher levels of negative emotions [69].

Americans generally follow news stories about public health, particularly disease-related stories [81]. To smokers, exposure to news stories reporting smoking disease raises awareness of a health risk and motivates preventive behavior [69]. Previous anti-smoking campaigns have utilized news media as a delivery vehicle of information encouraging smokers to quit [82, 83]. If reading a news story can bring about this desirable change for smokers, we conjecture that intervention messages with similar designs might also create such a positive change. Given the ubiquitousness of news media exposure, embedding intervention messages in news stories might be a way to increase informational coverage, which is important in anti-smoking campaigns using mass media [83, 84]. Our study heeds scholars’ call to provide initial evidence that smoking prevention campaigns might capitalize on the fear of COVID-19 to encourage smokers to make important steps toward quitting smoking [12, 13]. Moreover, our study demonstrates that to ensure this strategy of leveraging fear is impactful, the association between comparative optimism and disposition optimism should be examined and clearly understood before launching a campaign.

This study has several limitations. First, generalizability is limited by a convenience sample of US smokers. Second, we did not assess actual behaviors. Instead, we measured smoking quit intentions in the next 6 months. Given that behavioral intentions are the best predictor of actual behavior [85], it is plausible that exposure to combined COVID-19 and smoking risk messages might ultimately lead to behavioral change for smokers. Third, the behavioral intentions scale did not include other methods smokers might use to quit smoking, such as use of prescription medication, social support, or unaided quit attempts. Future studies can help to shed light on predicting these diverse quitting methods that smokers might use. Fourth, it should be noted that the study sample’s mean age suggested that participants were not likely in the high-risk group (*M* = 41; *SD* = 15.11). Thus, the result might be different if more participants at older age were recruited. Finally, COVID-19 has disproportional impacts on communities of different racial and gender groups [86, 87] and future studies should focus on risk factors among these at-risk and underserved populations.

In conclusion, our research responded to scholars’ call to turn the adversity of the COVID-19 pandemic into intervention strategies to control tobacco use, particularly at the early stages of the pandemic. Messages communicating the increased COVID-19 severity for smokers may motivate smokers’ intentions to take measures to quit smoking by evoking fear. Smokers with higher levels of comparative optimism are particularly likely to exhibit this relationship. As the pandemic continues, messages like these might serve as feasible strategies to combat the tobacco epidemic.

## Supporting information

S1 Appendix (DOCX)
