# Supplementary material for: Are smokers scared by COVID-19 risk? How fear and comparative optimism influence smokers’ intentions to take measures to quit smoking
Source: PLoS One. 2021 Dec 7;16(12):e0260478. doi: 10.1371/journal.pone.0260478 (PMC8651098; doi:10.1371/journal.pone.0260478)

**Appendix A**

Participant flowchart

**Appendix B**

1,004 current smokers

were randomized into 4 conditions

252 participants assigned to the COVID-19 risk exposure condition

252 participants assigned to the smoking risk exposure condition

243 participants assigned to the combined risk exposure condition

257 participants assigned to the control condition

495 participants were included in the analysis of the present study

Example of News Stimuli in the Smoking Risk Condition


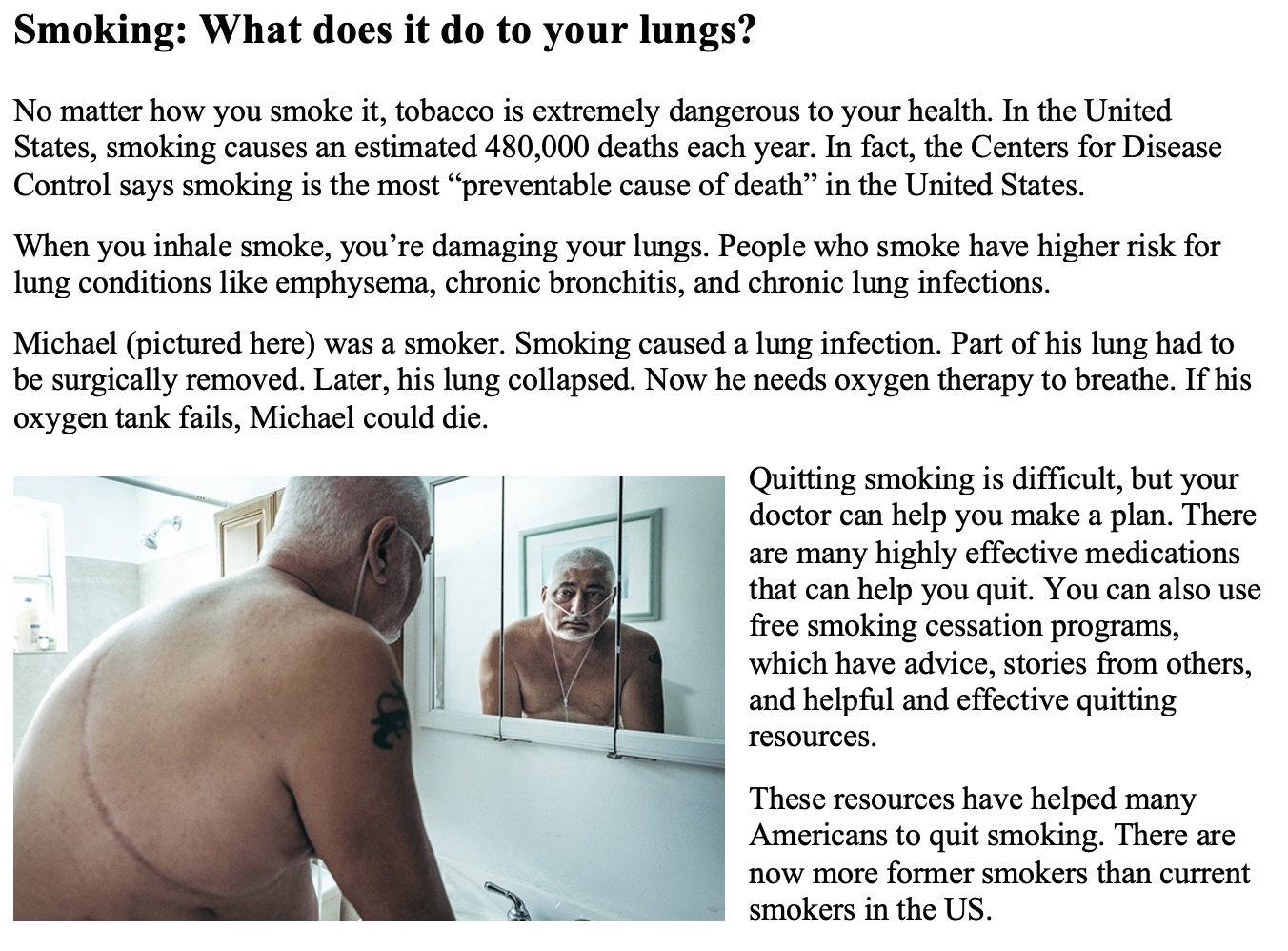


Image source: https://www.cdc.gov/tobacco/campaign/tips/resources/photos/index.html

Example of News Stimuli in the Combined Risk Condition


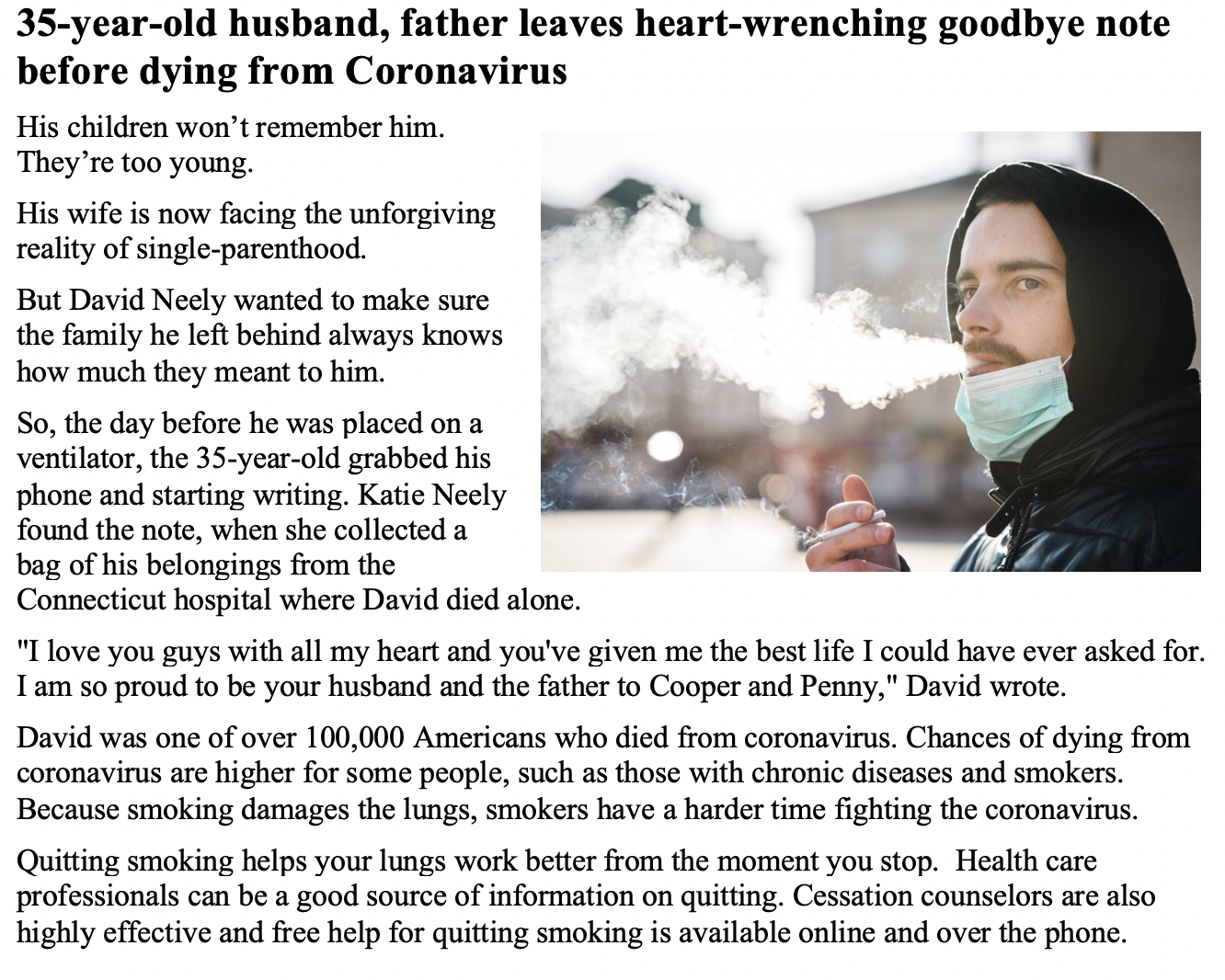

Supplement: S1 Appendix — (DOCX) [file pone.0260478.s001.docx]
